# Impact of Rye Kernel-Based Evening Meal on Microbiota Composition of Young Healthy Lean Volunteers With an Emphasis on Their Hormonal and Appetite Regulations, and Blood Levels of Brain-Derived Neurotrophic Factor

**DOI:** 10.3389/fnut.2018.00045

**Published:** 2018-05-29

**Authors:** Olena Prykhodko, Jonna Sandberg, Stephen Burleigh, Inger Björck, Anne Nilsson, Frida Fåk Hållenius

**Affiliations:** ^1^Food for Health Science Centre, Lund University, Lund, Sweden; ^2^Department of Food Technology, Engineering and Nutrition, Lund University, Lund, Sweden

**Keywords:** *Bacteroides*, *Prevotella*, *Faecalibacterium*, rye kernel bread, gut-brain axis

## Abstract

Rye kernel bread (RKB) evening meals improve glucose tolerance, enhance appetite regulation and increase satiety in healthy volunteers. These beneficial effects on metabolic responses have been shown to be associated with increased gut fermentation. The present study aimed to elucidate if RKB evening meals may cause rapid alterations in microbiota composition that might be linked to metabolic-, immune-, and appetite- parameters. Gut-brain axis interaction was also studied by relating microbiota composition to amount of brain-derived neurotrophic factor (BDNF) in blood plasma. Nineteen healthy volunteers, ten women and nine men aged 22–29 years, BMI < 25 (NCT02093481) participated in the study performed in a crossover design. Each person was assigned to either white wheat bread (WWB) or RKB intake as a single evening meal or three consecutive evenings. Stool and blood samples as well as subjective appetite ratings were obtained the subsequent morning after each test occasion, resulting in four independent collections per participant (*n* = 76). DNA was extracted from the fecal samples and V4 hypervariable region of the bacterial 16S rRNA genes was sequenced using next generation sequencing technology. Higher abundance of *Prevotella* and *Faecalibacterium* with simultaneous reduction of *Bacteroides* spp. were observed after RKB meals compared to WWB. The associations between metabolic test variables and microbiota composition showed a positive correlation between *Bacteroides* and adiponectin levels, whereas only *Prevotella* genus was found to have positive association with plasma levels of BDNF. These novel findings in gut-brain interactions might be of importance, since decreased levels of BDNF, that plays an essential role in brain function, contribute to the pathogenesis of several major neurodisorders, including Alzheimer's. Thus, daily consumption of *Faecalibacterium-* and/or *Prevotella*-favoring meals should be investigated further for their potential to prevent neurodegenerative processes in the brain.

## Introduction

Colonization of the intestinal tract with commensal microorganisms occurs immediately after birth, establishes during digestive and immune system maturation in early childhood and remains relatively stable in healthy adults (1, 2). The gut microbiota of each person is highly diverse among individuals, each representing a unique microbial ecosystem which can be referred to as an individual “microbiome fingerprint” (3). However, despite the relatively stable microbiome in a healthy population, alterations in colonic microbiota composition may occur relatively rapidly after intake of indigestible carbohydrates, i.e., dietary fibers. It is well-known, that dietary fibers in the colon can be used as substrates for fermentation process, depending on the individual fermentative capacity of different bacterial species (4–6). Furthermore, the end products of bacterial fermentation, such as short chain fatty acids (SCFAs), have been shown to exert beneficial effects on human energy metabolism (7).

Several dietary fibers have positive influence on metabolic responses in humans such as e.g. arabinoxylan, fructans (inulin), beta-glucans, and resistant starch (8–10). Wholegrain cereal constitutes one of the major sources of dietary fibers (11). Such fibers are associated with benefits on metabolism including reduced risk factor for type-2 diabetes and cardiovascular disease, i.e., via improved insulin sensitivity (12, 13). Moreover, semi-acute studies in healthy non-overweight participants showed that both barley (14) and rye (15, 16) kernel-based evening meals improved glucose tolerance and beneficially affected gut hormonal profiles, i.e., increased PYY, GLP-1, and GLP-2 and lowered free fatty acids (FFA) levels. In fact, the positive effects of barley kernel-based meals were strongly associated with colonic fermentation and also linked to alterations in the bacterial family *Bacteroidaceae*, specifically an increase in *Prevotella* and decrease in *Bacteroides* abundance (17). Although no differences in microbiota composition was reported in a Finnish adult cohort with metabolic syndrome after consumption of whole-grain and fiber-rich rye bread (18), information concerning potential changes in gut microbiome composition following rye ingestion as a means to prevent metabolic disturbances is absent. Functional changes of microbiota after rye kernel-based meals, such as increased concentrations of breath hydrogen and production of SCFAs and, especially, of butyrate and propionate, have previously been reported (15).

The main aim of the present study was to elucidate the impact of whole grain rye kernel evening bread-based meals (single occasion or three consecutive evenings) on the fecal microbiota composition in healthy lean volunteers. The objective was also to relate microbiota composition to glucose regulation, gut hormonal profile, inflammatory tonus, plasma SCFAs and also on subjective appetite ratings. In addition, in order to better understand gut-brain axis interactions, the relation of microflora composition to the amount of brain-derived neurotrophic factor (BDNF) in blood that plays an essential role in neuronal survival and synaptic plasticity in the brain was also studied in the same cohort of participants.

## Materials and methods

### Ethical statement

The study was registered at ClinicalTrials.gov (NCT02093481) and approved by the Regional Ethical Review Board in Lund, Sweden (Reference number 2013/241). All study participants gave written informed consent in accordance with the Declaration of Helsinki.

### Participants and study design

Twenty-five healthy lean volunteers (mean BMI ± SD: 21.9 ± 1.87 kg/m^2^) from Malmö-Lund region in Sweden were enrolled to participate in the study. In total, 19 non-smoker persons met the study criteria, i.e., being non-vegetarians, without any known metabolic disorders, not having gluten- or lactose- intolerance as well as food allergies. The average age of participants, including 9 men and 10 women, was 25.6 ± 3.5 years.

The study was performed in a crossover design where each person was served as his/her own control. All persons in the study were assigned to either white wheat bread (WWB) or rye kernel bread (RKB) intake as a single evening meal (referred to RKB 1d and WWB 1d) or during 3 consecutive days (referred to RKB 3d and WWB 3d), resulting in 4 independent test occasions for the same person (corresponds to 76 test occasions in total). The order of the product consumption was randomized, resulting in 12 different combinations, see supplementary Figure 1 for schematic illustration.

During the whole study, the participates were encouraged to maintain their regular eating habits without changing their meal pattern, since each subject acted as his/her own reference. Excessive physical exercise was not allowed 1 day prior to testing, while alcohol and fiber-rich foods were not allowed within 3-days period prior to the experimental day, hence also included period of bread consumption. Antibiotics or probiotics were not allowed during the whole study period and persons who consumed antibiotics during the last month prior to screening were not included in the study. More detailed information regarding exclusion criteria and experimental design can be found in Sandberg et al. (15).

### Samples collection and experimental procedure

The last portion of RKB and WWB before the experimental day was consumed at 9:30 p.m.; thereafter the study participants were fasting until the breakfast was served the following morning at the research unit at the Food for Health Science Centre at Lund University. Prior to breakfast, an intravenous cannula (BD Venflon™ Pro Safety Shielded IV Catheter, Becton Dickinson) was inserted into an antecubital vein by a nurse and fasting blood samples were collected, and breath H_2_ excretion were registered together with appetite questionnaire including subjective evaluation of satiety and desire to eat using a 100 mm Visual Analogue Scale (VAS).

At the experimental days, the overnight-fasted participants that arrived to the research unit in the morning were provided a standardized breakfast consisted a glass of water (200 ml) and a slice of WWB (121.4 g), which corresponded to 50 g available starch baked according to the same standardized procedure as the WWB meal (15). The standardized breakfast was served with request to be consumed within 15 min. Only low physical activity during the 3 h postprandial period was allowed. All persons collected stool sample themselves into the provided and labeled sterile tubes (Sarstedt, Nümbrecht, Germany) according to instructions. All fecal samples were obtained from the first stool at the experimental day, that corresponds to the end of the certain product consumption, and transported to the research unit within 30 min and stored at −80°C until analyses. In total, 76 stool samples were collected during the study.

### Rye kernel-based bread and reference white wheat bread

The test bread RKB was composed of 85% whole rye kernels and 15% white wheat flour, based on cereal dry matter. A refined wheat bread, WWB, consisting of 100% white wheat flour (based on cereal dry matter) was included as a reference for each subject. The size of the test portions, to be consumed in the evening, was based on 50 g available starch. The rye kernels (commercial blend) were provided by Lantmännen Cerealia (Järna, Sweden), and commercial white wheat flour was obtained from Kungsören AB (Järna, Sweden). The composition of carbohydrates for each of breads is shown in Table 1. The baking and storage procedure for the breads were described by Sandberg et al. (15).

**Table 1:** Carbohydrate composition of reference white wheat bread (WWB) and rye kernel bread (RKB).

**Carbohydrate composition**	**WWB**		**RKB**	
	**% DM**	**g/portion**	**% DM**	**g/portion**
Total dietary fiber	6.59	3.89	21.00	15.60
Total starch	80.50	51.30	66.60	53.90
Resistant starch	2.02	1.29	4.80	3.89
**Non-starch polysaccharides:**			
soluble	2.00	1.14	3.81	2.77
insoluble	2.57	1.47	12.30	8.98
Arabinoxylan	0.50	0.30	6.12	4.96
Fructan	0.50	0.30	4.00	3.24
Beta glucan	nd	nd	nd	nd

*The portion size was based on 50 g available starch. DM, dry matter; nd, not determined*.

### Metabolic parameters

Fasting and postprandial measurements, which totally lasted 3 h, were performed at all four visits. Briefly, the capillary blood from fingers was used for blood glucose measurements (HemoCue®B-glucose, HemoCue AB, Ängelholm, Sweden). Breath hydrogen was measured as an indicator of colonic fermentation, using an EC60 gastrolyzer (Bedfont EC60 Gastrolyzer, Rochester, England). Venous blood was collected to determine serum levels of insulin, FFA, CRP, IL-6, IL-18, adiponectin, PAI-1, triglycerides (TG), while plasma was used to measure GLP-1, PYY, ghrelin, GLP-2, nesfatin-1, and SCFA as described by Sandberg et al. (15). The level of BDNF in plasma was measured by enzyme immunoassay kit (ChemiKine BDNF Sandwich ELISA kit, CYT306, Millipore Bioscience Research Reagents, USA and Canada), while plasma SCFAs (acetate, propionate and butyrate) were analyzed using a gas-chromatography method (19).

### Fecal microbiota

Extraction of DNA was performed from 50 to 100 mg of fecal sample using the QIAamp DNA Stool Mini Kit (Qiagen, Hilden, Germany), according to the manufacturer's protocol using an additional bead-beating step to improve lysing of the cells. Measurement of DNA concentration was performed using Qubit 2.0 Fluorometer (Thermo Fisher Scientific). Then DNA was amplified by polymerase chain reaction (PCR) using the primers for the target gene 16S rRNA (hypervariable V4 region) were 515F 5′-GTGCCAGCMGCCGCGGTAA-3′ and 806R 5′-GGACTACHVGGGTWTCTAAT-3′ for forward and reverse reads, respectively (20). Paired-end sequencing with a read length of 2 × 250 bp using Miseq V2 reagent kit (Illumina) was carried out on a Miseq DNA sequencer (Illumina Inc., San Diego, USA). Illumina sequencing adaptors were trimmed off during the generation of FASTQ files and reads that did not match any barcodes were discarded.

The amplicon library preparation and sequencing procedure were performed for all fecal samples at once (*n* = 76), minimizing the “batch-effect”. However, the result from the two test occasions was excluded for one subject due to the acute respiratory infection, giving only 18 observations for WWB 1d and RKB 1d treatment in the studied cohort. Therefore, 74 sequenced samples (WWB 1d, *n* = 18; WWB 3d, *n* = 19; RKB 1d, *n* = 18; RKB 3d, *n* = 19) were further proceeded for bioinformatics using the open-source bioinformatic pipeline, Quantitative Insights into Microbial Ecology (QIIME). In brief, forward and reverse reads were joined and then quality filtering was performed. In total, 10,720,780 reads were used for 74 samples with a mean of 144,875 reads per samples, (min: 28,754 and max: 275,138). In addition, as an alternative step to the already established pipeline, the quality of individual forward and reverse reads was rechecked prior to the joining step, resulting in similar values, such as 11,014,566 of total reads, 148,845 as a mean, and 29,686 and 278,873 as a min and max reads per sample, respectively. The sequences were grouped into operational taxonomic units (OTUs) at a minimum of 97% similarity by using the closed reference method based on the Greengenes database (v.13.8), resulting in similar percentage of assigned bacteria between tested pipelines. After filtering of singletons and low abundance OTUs (< 0.0001), analysis of alpha diversity (alpha rarefaction, Shannon and Simpson indices) as well as beta diversity of the microbial community were applied to the OTU table at an even depth of 28,000 sequences per sample, thus including all samples in the analysis. Prediction of functional capacity of microbiota (PICRUSt) and algorithm for high-dimensional biomarker discovery (LEfSe) was also included in the pipeline using Galaxy bioinformatics open platform (GitHub, www.github.com/galaxyproject) see supplement for detailed information.

### Calculations and statistical methods

Data regarding relative abundances of fecal microbiota were grouped due to test occasion irrespective of the visit order and expressed as means ± SEM for each group (WWB 1d, *n* = 18; WWB 3d, *n* = 19; RKB 1d, *n* = 18; RKB 3d, *n* = 19), where comparison of duration (1d vs. 3d) or type of bread consumed (WWB vs. RKB) was evaluated. Note, that correlation of the metabolic parameters and subjective appetite ratings was done grouping data from individual person, resulted in total 74 XY observations for each studied parameter (2 XY observations were excluded for one subject due to respiratory infection during two test-occasions as described above).

Statistical evaluations involving glucose and insulin concentrations are based on incremental changes from fasting concentrations. The absolute values are used in the statistical calculations of the rest of the test variables. The incremental area under the curve (iAUC_0−120_) was calculated for each subject and test meal, using the trapezoid model. GraphPad Prism (version 7, GraphPad Software, San Diego, CA, USA) was used for graphs plotting and for Pearson correlation. Significant differences in bacterial operational taxonomic units (OTU) were assessed with ANOVA or/and paired *t*-test (the Bonferroni correction was applied to correct for multiple testing). The differences were considered to be significant if *p* < 0.05, while a trend if *p*-value levels ranged from > 0.05 to ≤ 0.08.

## Results

**Figure 1:**
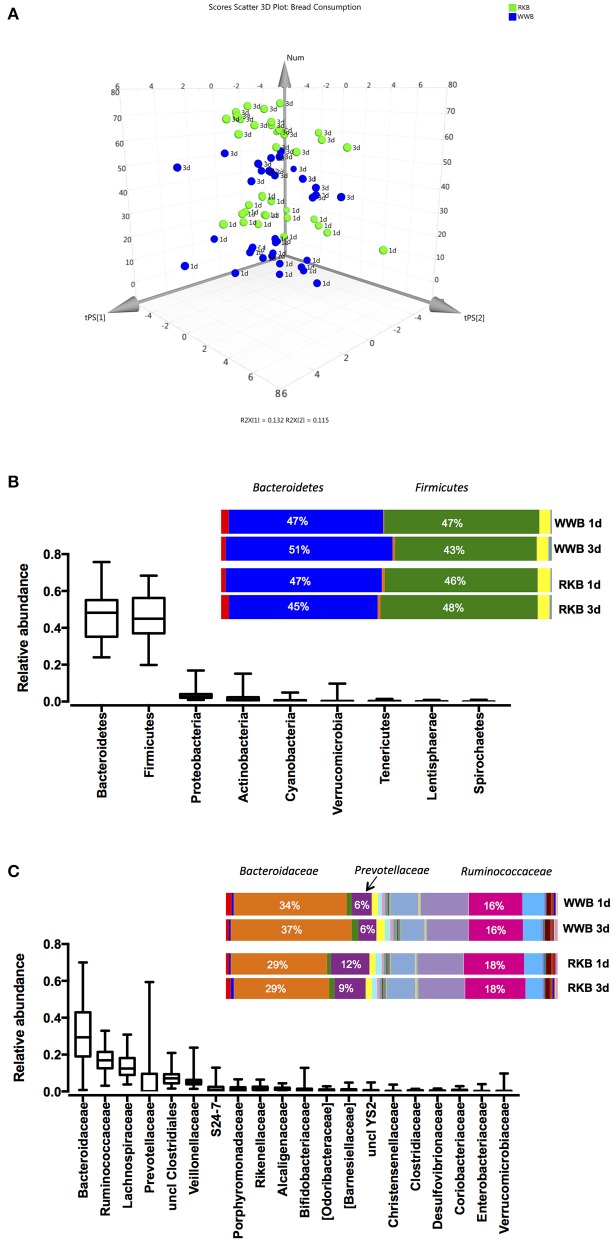
3D PCA plot of data points for each participant after WWB and RKB product consumption during 1 or 3 d period **(A)**; Relative abundance of OTUs taxonomically assigned to bacterial phyla **(B)** and family **(C)** level, of all participants at all visits (unfilled boxplots, *n* = 74) or per test occasion (colored bars), where RKB 1d (*n* = 18), WWB 1d (*n* = 18), RKB 3d (*n* = 19), WWB 3d (*n* = 19) WWB, white wheat bread; RKB, rye kernel bread.

The general distribution of the samples after the principal component analysis, that included sequenced data of fecal microbiota per each participant, showed clustering of the study participants after RKB or WWB product consumption at one occasion in the evening or following three consecutive evenings (Figure 1A). The analysis of bacterial 16S RNA gene sequences showed that nine bacterial phyla were observed, the most abundant being Bacteroidetes (48%) and Firmicutes (46%) followed by Proteobacteria (3%), Actinobacteria (2%), Cyanobacteria (1%), Verrucomicrobia (0.5%), Tenericutes (0.2%), Lentisphaerae (0.1%), and Spirochaetes (< 0.01%). The bacterial community at the phylum level was not evidently changed after RKB or WWB meal consumption (Figure 1B). The OTUs assigned taxonomically to family level resulted in 37 families, the 20 most abundant being shown in Figure 1C, although only three were dominating, represented by *Bacteroidaceae* (32%), *Ruminococcaceae* (17%), and *Lachnospiraceae* (14%). It is worth mentioning that the *Prevotellaceae* family showed the highest variation within the cohort, ranging from 0.003 to 59%. The differences in bacterial composition after intake of RKB were more evident on this taxonomical level, shown lower abundance of members from *Bacteroidaceae* family with concomitant higher of *Prevotellaceae* and *Ruminococcaceae* compared to WWB (*p* < 0.05), independently on duration period.

**Figure 2:**
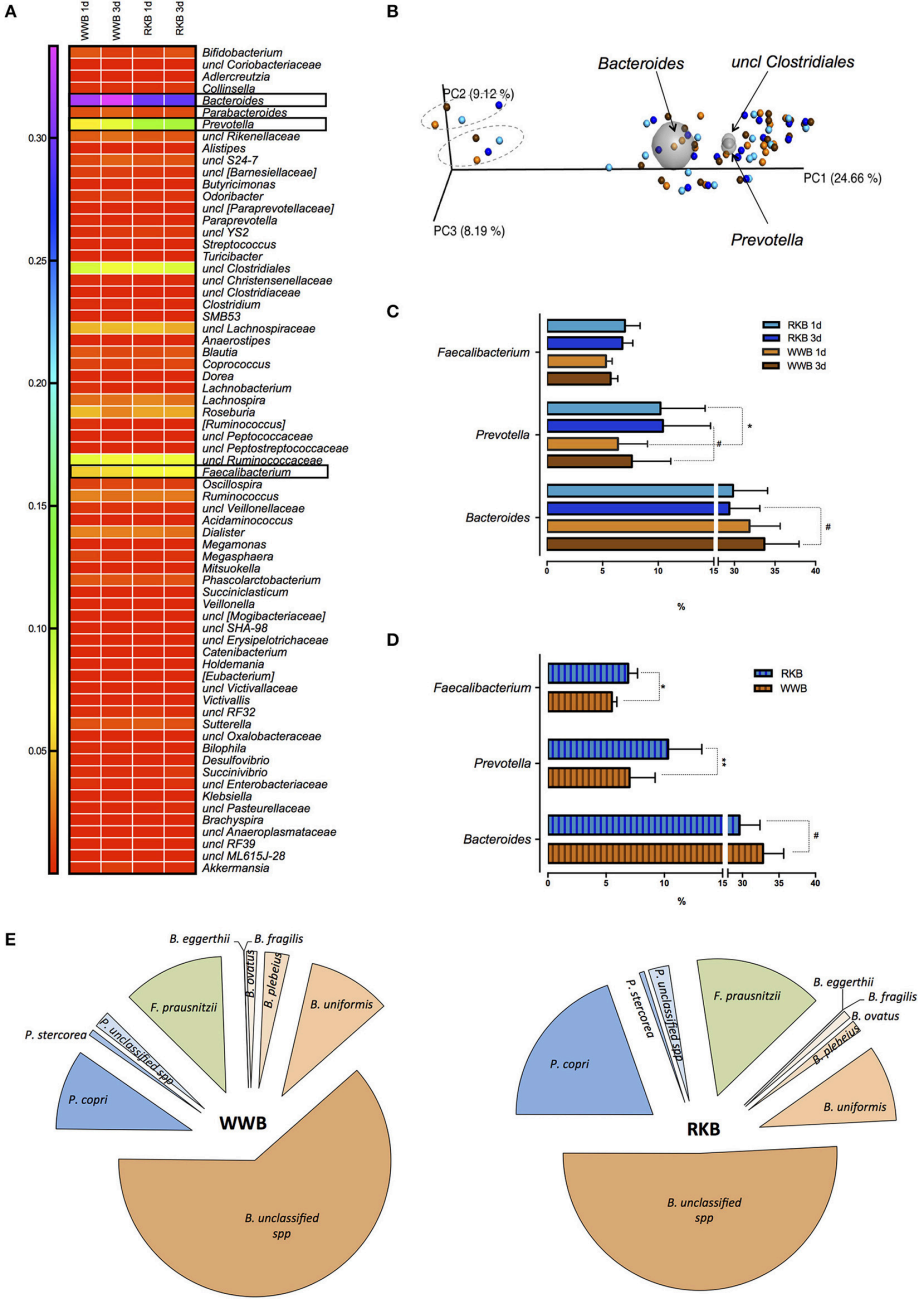
The OTUs assignment to the genus level presented as a heat map per each test occasion after WWB and RKB product consumption during 1 or 3d period **(A)**; PCoA plot showing distribution of bacterial community between the participants after consumption of RKB 1d (light blue circles), RKB 3d (blue circles), WWB 1d (orange circles) and WWB 3d (brown circles), while bi-plots (grey spheres) visualize the three taxa driving the differences in the cohort where outlier two persons are circled with dashed line **(B)**; The most significant differences in bacterial genera between bread consumption for each test occasions included duration of the bread consumption **(C)** where RKB 1d (*n* = 18), WWB 1d (*n* = 18), RKB 3d (*n* = 19), WWB 3d (*n* = 19), and pooled result **(D)** from each type of bread (RKB vs. WWB, *n* = 37/per each bread) on genus level or **(E)** species level. *p < 0.05, **p < 0.01, ^#^*p* ≤ 0.08; RKB, rye kernel bread; WWB, white wheat bread; *B., Bacteroides*; *P., Prevotella*; *F., Faecalibacterium*; spp., species.

The OTU assignment to genus level resulted in 69 different genera as presented by a heat map with three genera, *Bacteroides, Prevotella*, and *Faecalibacterium* consistently responding to the product consumption (Figure 2A). The most shared genera between all samples were *Bacteroides, Prevotell*a and an unclassified genus of order *Clostridiales* as shown by principal component analysis of beta-diversity with the exception of two participants in the present cohort (Figure 2B). The samples showed normal distribution, even though no significant differences in *alpha-* (including Shannon and Simpson indices) or *beta-* diversity were observed between treatments. Further statistical comparison of microbial genera of volunteers ingesting RKB or WWB showed significantly higher abundance of *Prevotella* genus after RKB 1d vs. WWB 1d (*p* < 0.05), whereas only tendency for *Prevotella* to increase and *Bacteroides* to decrease, was observed after 3 days period consumption of RKB compared to WWB with *p* = 0.08 and *p* = 0.06, respectively (Figure 2C). Pooled data from product intake, disregarding the consumption duration, resulted in significant increase of *Prevotella* (*p* < 0.01), and *Faecalibacterium* (*p* < 0.05), which coincided with tended decrease of *Bacteroides* (*p* = 0.06) after RKB consumption in comparison to WWB (Figure 2D). The relationship between *Prevotella, Bacteroides*, and *Faecalibacterium* resulted in a strong negative correlation (*r* = −0.632, *p* < 0.0001) between *Prevotella* and *Bacteroides*, while no significant relation was obtained between these genera and *Faecalibacterium*. In addition, the relative abundance of assigned bacteria to species level showed 2-fold increase in *P. copri* abundance but neither increase of *P. stercorea* nor unclassified species of *Prevotella* were observed. Abundance of *F. prausnitzii*, which is the only *Faecalibacterium* species, also was higher after RKB consumption compared to WWB (*p* < 0.05) (Figure 2E). Although generally lowering effect of RKB ingestion was observed on *Bacteroides* genus, including *B. eggerthii, B. fragilis, B. ovatus, B. plebeius*, and *B. uniformis*, the most evident decrease was observed for unclassified species of *Bacteroides* genus.

**Figure 3:**
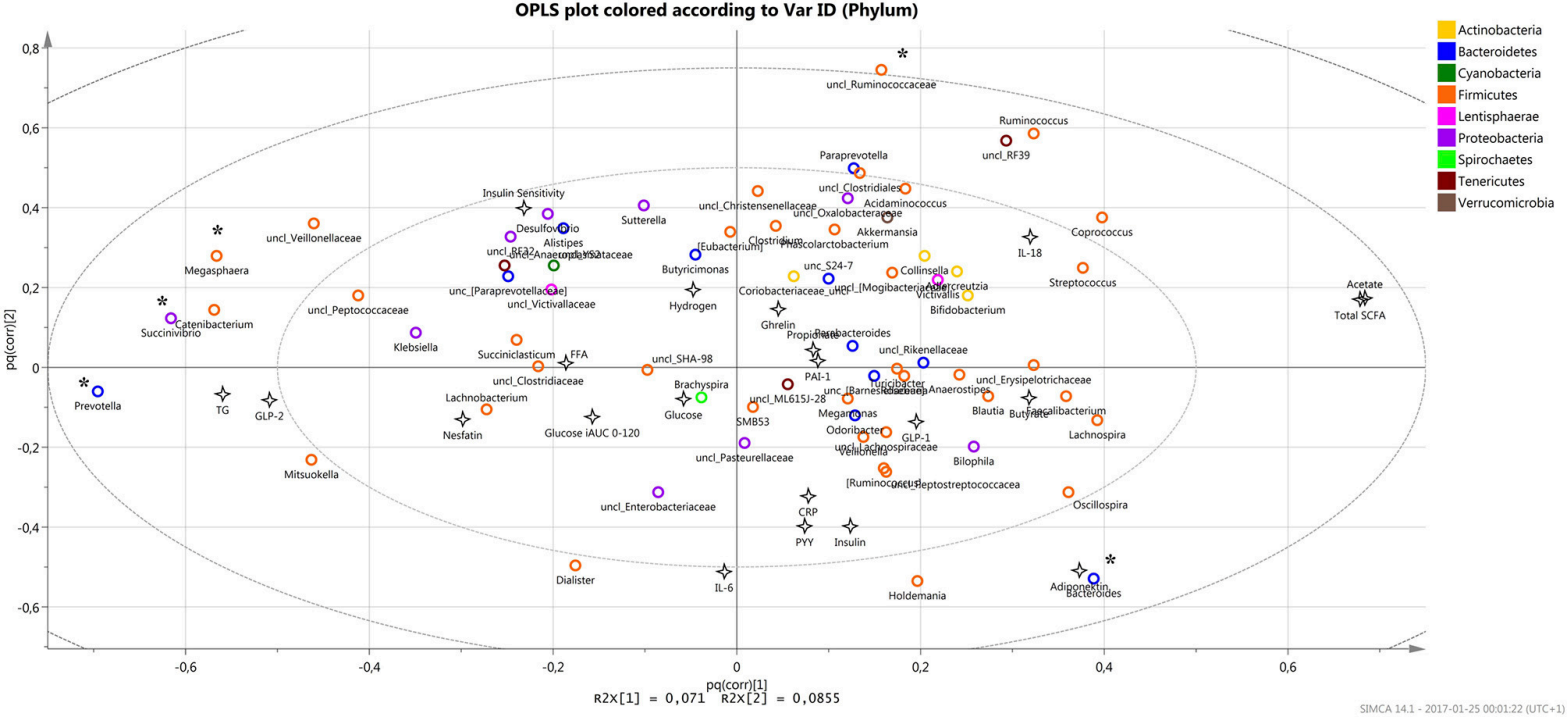
The OPLS plot displays the relation between all X and Y variables in the study (max 74 XY par for each investigated parameter) where the most significant bacteria as X-variables are situated away from the center on the positive or negative side of the plot, with the most significant variables that are found on the periphery (marked *). X and Y variables opposite to each other are negatively correlated while positively correlated variables situated near each other.

Relations between metabolic parameters that have been previously reported by Sandberg et al. for the current cohort (15) together with current observations in microbiota composition were studied using SIMCA (v.14) prediction program for multivariate analysis (UMETRICS, Umeå, Sweden). First, the most significant X-variables of importance for the projection (VIP) were found with the genera that exceed VIP value >1. In total, 18 genera from Firmicutes phylum, 6 from Bacteroidetes, 3 from Proteobacteria, 2 from Tenericutes and 1 from Lentisphaerae were identified to be most important in the prediction system with *Prevotella, Megasphaera, Succivibrio*, unclassified *Ruminococcaceae*, and *Bacteroides* as a top five. Orthogonal partial least squares (OPLS) algorithm was applied for the system to show covariance between X (microbiota on genus level) and Y (metabolic and hormonal parameters) resulted in distributing the top five mentioned genera on the periphery of the obtained plot (Figure 3). The result showed a strong positive correlation between *Bacteroides* and adiponectin (*r* = 0.4; *p* = 0.0004), while *Prevotella, Megasphaera, Succivibrio* were found to have negative correlation with blood acetate and total level of SCFA (*p* < 0.05). In addition, the unclassified genera of *Ruminococcaceae* family showed negative correlation with fasting levels of IL-6 cytokine (*p* = 0.039) and adiponectin (*p* = 0.007).

We also verified if the quality parameters are stable toward permutation (randomization) test to find important Y-variables for our model. This test showed that FFA, fasting glucose, glucose iAUC_0−120min_, insulin and butyrate were found to be the least reliable blood parameters in the prediction model than others and will therefore not be discussed further in the present paper.

Each overnight-fasted subject answered the questionnaires, regarding subjective appetite parameters (using VAS, Visual Analogue Scale criteria for desire to eat and satiety) at the experimental day that is also corresponds to the day of the stool collection. The results from the appetite regulation, including gut hormones profiles, and also subjective ratings of satiety, hunger and desire to eat have been previously published for the present cohort in form of original values (15). Here we used data such as satiety and desire to eat in fasting conditions only in order to estimate if there are any associations with fecal microbiota profiles. Thus, the correlations between bacterial genera and these appetite feelings were estimated and only those that were found to be significant are shown (Table 2). In total, 19 different genera found to be related to at least one feeling, from which 5 showed relation to both, desire to eat and satiety. For instance, unclassified genus of *Anaeroplasmataceae* family, as well as *Lachnospira* genus negatively correlated with desire to eat and positively with satiety, while unclassified family and genus of *Clostridiales* order as well as *Parabacteroides* and unclassified *SHA-98* positively correlated with desire to eat and negatively with satiety.

**Table 2:** Pearson correlation of bacterial genera and subjective feelings of appetite.

**Pearson correlation**		**Desire to eat**				**Satiety**	
	**r**	***R^2^***	**P-value**		***r***	***R^2^***	**P-value**	
*Holdemania*	−0.36	0.131	0.002	^**^	0.15	0.024	0.199	
*[Ruminococcus]*	−0.33	0.110	0.005	^**^	0.08	0.007	0.499	
*Bacteroides*	−0.29	0.084	0.014	^*^	0.23	0.051	0.058	
*uncl Anaeroplasmataceae*	−0.27	0.072	0.024	^*^	0.7	0.224	< 0.0001	^****^
*Oscillospira*	−0.26	0.068	0.029	^*^	−0.02	0.000	0.897	
*Lachnospira*	−0.25	0.063	0.035	^*^	0.24	0.057	0.045	^*^
*uncl [Paraprevotellaceae]*	−0.19	0.036	0.113		0.34	0.118	0.003	^**^
*uncl Peptococcaceae*	−0.12	0.014	0.318		0.29	0.086	0.013	^*^
*Paraprevotella*	0.10	0.009	0.429		−0.24	0.059	0.041	^*^
*Veillonella*	0.13	0.017	0.276		−0.25	0.061	0.038	^*^
*Clostridium*	0.15	0.022	0.220		−0.25	0.064	0.033	^*^
*Acidaminococcus*	0.15	0.022	0.214		−0.30	0.091	0.010	^*^
*uncl Ruminococcaceae*	0.16	0.027	0.173		−0.24	0.058	0.043	^*^
*Desulfovibrio*	0.21	0.045	0.076		−0.31	0.095	0.009	^**^
*Turicibacter*	0.24	0.057	0.046	^*^	−0.14	0.018	0.260	
*uncl Clostridiales*	0.26	0.065	0.032	^*^	−0.32	0.104	0.006	^**^
*Parabacteroides*	0.28	0.081	0.016	^*^	−0.27	0.071	0.025	^*^
*uncl [Barnesiellaceae]*	0.33	0.106	0.006	^**^	−0.20	0.039	0.100	
*uncl SHA-98*	0.34	0.115	0.004	^**^	−0.25	0.064	0.033	^*^

Significance indicated as

* *p < 0.05*,

** *p < 0.01*,

**** *p < 0.001 for fasting conditions*.

**Figure 4:**
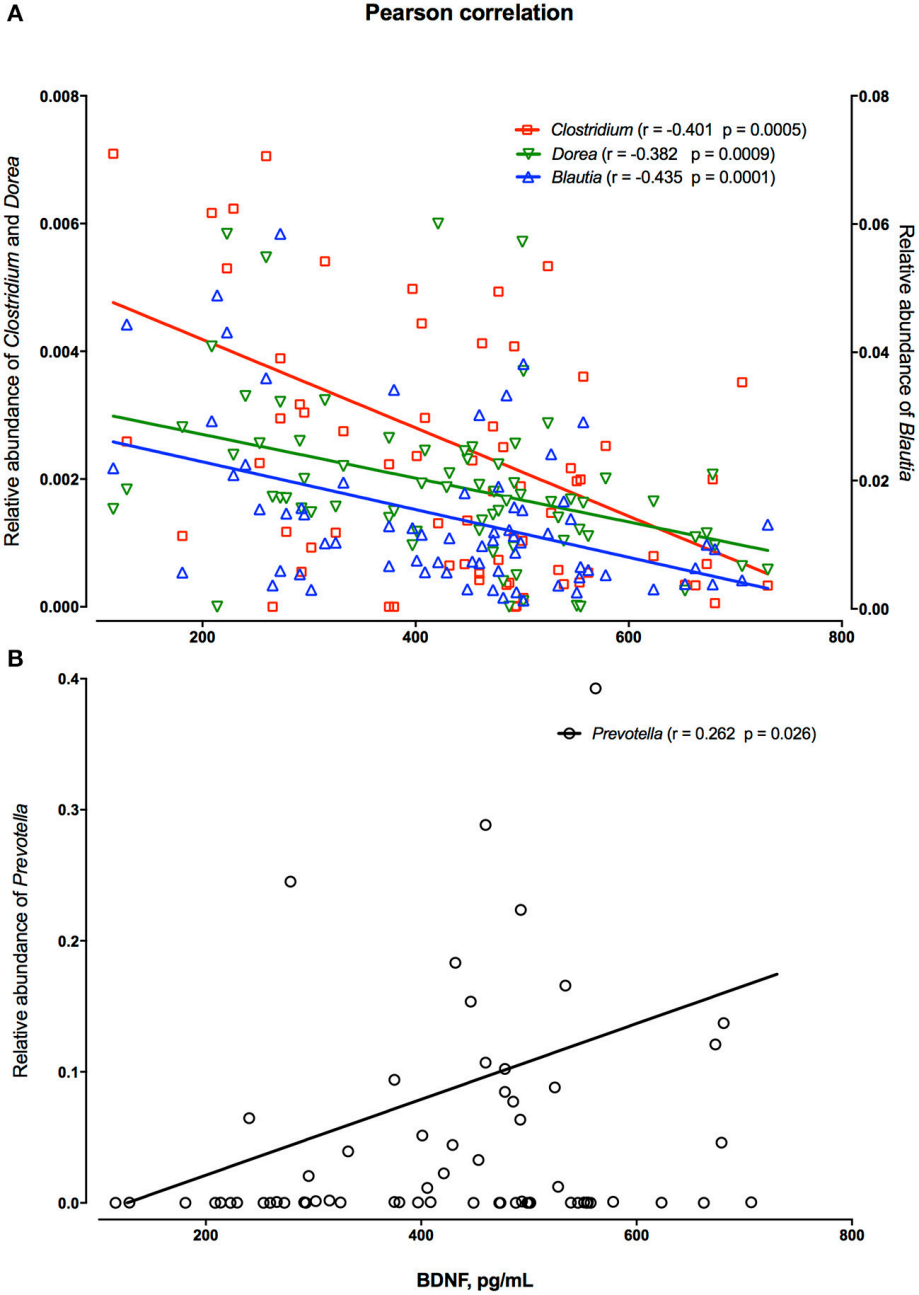
Pearson correlation between relative abundance of fecal *Clostridium, Dorea, Blautia* **(A)** and *Prevotella* of individual persons irrespectively of the product consumption or priming period **(B)** and their blood levels of brain-derived neurotrophic factor (BDNF).

The relation of bacteria to levels of brain-derived neurotropic factor (BDNF) showed that genera *Clostridium, Dorea* and *Blautia* were negatively correlated with blood levels of BDNF (Figure 4A), while positive correlation of BDNF in blood was observed for the *Prevotella* genus only (Figure 4B).

The results from the functional capacity of microbiota, using PICRUSt metagenome prediction software, showed significantly higher number of bacterial OTUs associated with circulatory system function (4814 vs. 440, *p* = 0.0013) and cardiovascular disease risk (1593 vs. 136, p = 0.0005) in men compared to women, while no other differences were observed between genders neither between test products nor priming lengths or product consumption order.

## Discussion

The main objective of the present study in healthy volunteers was to investigate changes in microbiota composition after 1d or 3d ingestion of rye kernel based bread (RKB) consumption in comparison to WWB. Further, efforts were made to study the potential relation between the fecal microbiota and metabolic test variables, subjective feelings of appetite, and blood level of BDNF.

The present study was designed to study the dynamic response of microbiota composition after consumption of both types of bread during 1 and 3 consecutive days by the same participant, with a 1-week washout period between the test occasions. Moreover, since each person was always served as its own control and product consumption order was randomized, resulting in 12 different combinations, the collection of the stool samples at baseline was omitted. Although, it has been shown that intake of WWB up to 3 days has no significant impact on microbiota composition (17) and thus can serve an appropriate baseline, the absence of samples of the initial microbiota composition in the present cohort limits our discussion only to comparison of two tested products.

### Shift in microbiota after rye kernel bread intake

The results showed that intake of RKB during 1 or 3 days priming affect microbiota alteration similarly, while 3-days priming with WWB ingestion affected mainly *Bacteroidaceae* family with higher abundance of *Bacteroides* spp. presumably due to wheat starch content in this product (21, 22). Despite the duration period, the RKB consumption promoted a rapid change of microbiota compared to WWB by favoring abundance of *Prevotellaceae* family with higher abundance of *Prevotella* spp. particularly *P. copri*, with simultaneous reduced abundance of all observed species of *Bacteroides*. Moreover, intake of RKB resulted in higher abundance of beneficial butyrate-producing *Faecalibacterium* represented by only known species of this genus — *F. prausnitzii* (21, 23). It is worth mentioning, that *Prevotella-*dominating microflora was recently found to have different capacity to utilize fiber, increasing colonic propionate- and butyrate- productions (24). It can be hypothesized that increased blood fasting levels of both butyrate and propionate after short duration of RKB intake, which has been previously reported for the present cohort (15), presumably linked to shift in *Prevotella*/*Bacteroides* abundance seen in the present study. We assumed that rapid changes in microflora composition were provoked by high consumption of total dietary fiber and particularly arabinoxylan and fructan, which was 7.5-folds and 6.8-folds, respectively, higher in RKB portion than in WWB (22). In addition, the effects of beta glucan on observed microbiota changes cannot be excluded, however we did not measure the amount of beta glucan in the tested breads, which limits our discussion regarding glucan intake in the present study.

### Fecal microbiota in relation to hormonal and appetite regulation

It has been previously shown for this particular cohort of volunteers that their subjective ratings of satiety feeling after RKB consumption in comparison to WWB were increased while subjective feelings of desire to eat were reduced at both, fasting conditions and after the standardized breakfast, disregarding the length of RKB priming period of 1 or 3 days (15). Moreover, these subjective appetite ratings were concomitant with increased satiety hormone such as PYY (15).

Although no associations between appetite-related gastrointestinal hormones such as ghrelin and PYY and abundance of specific bacteria were found in present study, the questionnaire regarding volunteers' subjective rating of appetite feelings at fasting conditions showed some associations with their gut microbiota. For instance, the strongest association of satiety after overnight fasting was observed with the *Anaeroplasmataceae* family in the present cohort. It is worth mentioning that the link between *Anaeroplasmataceae* and higher subjective satiety has been already reported (25, 26). In contrast to satiety, the positive correlation of desire to eat in our study was found with unclassified order *SHA-98*. This bacterium has been primarily reported in lean population (BMI < 25) in strong association with their genetic profiles (27, 28). Although our study showed a clear impact of RKB on certain bacteria in lean volunteers together with published data that wholegrain rye meals have a significant impact on appetite regulation (15, 16), there is not enough knowledge to further discuss links between subjective appetite parameters and microbiota composition, since factors unrelated to the diet, i.e., genetic background, might be involved here and, hence, need to be separately explored. In addition, since the present study was aimed to investigate relations of microbiota composition to subjective rating of appetite feelings in lean persons at fasting conditions only, future work would also include standardized measurements of energy intake during *ad libitum* lunch together with appetite sensations to further strengthen the obtained results.

In the present study, we found a positive correlation between *Bacteroides* and adiponectin blood levels, which can be linked to intake of resistant starch (29). Other links between metabolic regulation and changes in microbiota composition were not observed in the present study possibly due to relatively low number of participants. On the other hand, since only healthy, young, lean and non-smoking persons participated in the study, their microbiota composition was found to be relatively stable throughout the whole study as seen by sequenced data from stool samples collected at four occasions separated in time, giving us the opportunity to investigate direct effect of the tested breads on the gut microbiota (4, 30). With this assumption, *Bacteroides* and *Prevotella* were found significantly reacting in response to the tested bread. It is not surprising that the prominent effect of tested bread meals were observed mainly on these most shared genera in the present cohort of healthy adults followed nordic diet recommendations (31).

### Gut-brain axis

It is worth mentioning that beneficial effects of whole grain-based, fiber rich diet on cognition have been associated with increased abundance of colonic butyrate producing bacteria (32, 33). In fact, the present study showed significant higher abundance of *Faecalibacterium prausnitzii* after RKB consumption in comparison to WWB, which is recognized as one of the most important butyric acid producing bacteria (23). Furthermore, in the present work the increased abundance of *Prevotella* spp. was found to have positive association with blood BDNF, a factor that is crucial for cognitive performance including learning and memory formation (34–36). It has been previously reported that BDNF is negatively correlated with *Clostridium* (37), which is also seen in our cohort, but negative correlation of BDNF with *Dorea* and *Blautia* is reported for the first time. Noteworthy, *Clostridium* and *Blautia* have been significantly increased in abundance in patients with major depressive disease, while *Prevotella* decreased (37).

The novel data, regarding relation of BDNF to microbiota must be further explored, since decreased levels of BDNF are associated with the pathogenesis of several major diseases including depression and Alzheimer's disease (35, 36, 38, 39), while daily consumption of meals favoring *Faecalibacterium prausnitzii* and/or *Prevotella* might possibly prevent or slow neurodegenerative processes, including dementia. The present data suggest possible effects of RKB on cognitive functions in healthy volunteers but, since cognitive tests were not performed for this particular cohort, further studies need to be performed as a proof of the concept.

### Functional capacity of the fecal microbiota

Despite that our study was done in a small cohort of healthy volunteers, the prediction of the functional capacity of the microbiota showed gender differences with regards to bacterial genes related to the disease development. For instance, the number of bacterial OTUs associated with function of circulatory (cardiovascular) system and risk for cardiovascular disease was found to be higher in men compared to women. Gender differences in risks for cardiovascular disease development were previously reported in relation to hormonal and biochemical profiles between man and woman (40). Thus, the present findings regarding microbiota function might be of importance, suggesting that bacterial profiles should be also taken into consideration, in order to predict possible development of risk factors.

## Conclusion

In conclusion, *Prevotella* genus was found to have positive association with blood levels of BDNF in a cohort of healthy young volunteers, whereas intake of rye kernel-based meals rapidly stimulated increase of fecal *Faecalibacterium prausnitzii* and *Prevotella copri* with simultaneous decrease of *Bacteroides* species.

## Author contributions

JS, AN, and IB conceived and designed the human trial. JS performed the experiments and samples collection. OP and SB performed microbiome sequencing and bioinformatics analysis. OP and FF results interpretation. OP wrote the manuscript draft. The paper was revised and approved by all authors.

### Conflict of interest statement

The authors declare that the research was conducted in the absence of any commercial or financial relationships that could be construed as a potential conflict of interest.
