# Supplementary material for: Impact of Rye Kernel-Based Evening Meal on Microbiota Composition of Young Healthy Lean Volunteers With an Emphasis on Their Hormonal and Appetite Regulations, and Blood Levels of Brain-Derived Neurotrophic Factor
Source: Front Nutr. 2018 May 29;5:45. doi: 10.3389/fnut.2018.00045 (PMC5986961; doi:10.3389/fnut.2018.00045)
Supplement: Supplementary file 1 [file Data_Sheet_1.docx]

Supplementary Material

**Impact of Rye Kernel-Based Evening Meal on Microbiota Composition of Young Healthy Lean Subjects With an Emphasis on Their Hormonal and Appetite Regulations, and Blood Levels of Brain-Derived Neurotrophic Factor**

Olena Prykhodko^1,2^ *, Jonna Sandberg^1,2^, Stephen Burleigh^1,2^, Inger Björck^1^, Anne Nilsson^1,2^, Frida Fåk Hållenius^1,2^

^1^ Food for Health Science Centre, Lund University, Lund, Sweden

^2^ Dept of Food Technology, Engineering and Nutrition, Lund University, Lund, Sweden

*Corresponding author: [olena.prykhodko@food.lth.se](mailto:olena.prykhodko@food.lth.se)

# Supplementary Data

**1.1 Bioinformatics tools summary**

Miseq fastq.gz files were gathered using a Python3 script and the associated mapping file validated by Qiime's validate_mapping_file.py.

FastQC was used to assess the quality of the Miseq reads and determine the Prinseq filtering parameters for the following step.

Based on the FastQC read quality analysis, Prinseq was used to trim read ends (L10, R7), select min and max read lengths (220 to 290) and filter based on a minimum QSCORE of 25.

Qiime's join_paired_ends.py was used to join the filtered forward and reverse Illumina reads.

The joined reads were used as input for Qiime's split_libraries.py, which had a filtering QSCORE set at 25.

After RDP classification the taxonomic hierarchies were charted using Krona.

Prinseq was also used to assess read quality of the trimmed reads.

Qiime's pick_closed_reference_otus.py was used to assign the reads to OTUs using uclust with the reverse-strand match option, a similarity score set at 0.97 and using the Greengenes core reference alignment (gg_13_8_otus rep_set). The input files were processed individually, therefore OTU assignments for each sample were independent from each other.

After picking, the Otus were filtered using Qiime's filter_otus_from_otu_table.py to remove singletons and low abundance Otus (minimum count fraction was set at 0.0001). After filtering, the individual sample biom files were merged using Qiime's merge_otu_tables.py. Biom format was used for both merging of the biom files and their summarization.

Qiime's group_significance.py was used to statistically compare OTU frequencies in sample groups using ANOVA. Qiime's group_significance.py was also used to compare functional predictions made by Picrust's categorize-by-function output. Qiime's group_significance.py was also used to compare pathway abundance predictions made by Humann2.

Emperor was used to make three-dimensional PCoA plots of weighted and unweighted coordinate files derived from Qiime's core_diversity_analyses.py.

Qiime's compare_categories.py was used to analyse the strength and statistical significance of groupings using ANOSIM.

Qiime's summarize_taxa_through_plots.py was used to summarize the taxonomic profiles derived from the filtered OTU biom file. This program was also used to summarize functional predictions from Picrust's categorize-by-function analysis.

Qiime's summarize_taxa_through_plots.py table files were used as input for MetaPhlAn2s metaphlan_hclust_heatmap.py to produce heat maps of taxonomic abundance for the various treatments.

Python3 together with matplotlib-venn was used to make Venn diagrams of taxonomic table outputs from Qiime's summarize_taxa_through_plots.py.

Graphlan graphs were made using Qiime's summarize_taxa_through_plots.py taxonomic summary files.

Python3 was used to convert Qiime's summarize_taxa_through_plots.py taxonomic assignments to a format suitable for R-based taxonomic analyses.

R was used for the statistical analysis of the taxonomic assignments derived from Qiime's summarize_taxa_through_plots.py, including boxplots and bar graphs of the taxonomic summaries.

The Lefse pipeline (format_input.py, run_lefse.py, plot_res.py, plot_cladogram.py) was used to generate LDA plots and cladograms based on the reformatted Qiime's summarize_taxa_through_plots.py taxonomic summaries.

Picrust's normalize_by_copy_number.py, predict_metagenomes.py, categorize_by_function.py with the KEGG_Pathways option was used to predict metagenome functional content from the taxonomic assignments provided by summarize_taxa_through_plots.py.

Qiime's summarize_taxa_through_plots.py was used to summarize the taxonomic profiles derived from the filtered OTU biom file. This program was also used to summarize functional predictions from Picrust's catagorize-by-function analysis.

Graphlan graphs were made using Picrust's functional assignments (categorize-by-function) that had been summarized by Qiime's summarize_taxa_through_plots.py.

Qiime's group_significance.py was used to statistically compare OTU frequencies in sample groups using ANOVA. Qiime's group_significance.py was also used to compare functional predictions made by Picrust's categorize-by-function output. Qiime's group_significance.py was also used to compare pathway abundance predictions made by Humann2.

Humann2's humann2_split_table was use to reformat Picrust's metagenome functional predictions output for Humann2 analyses.

Graphlan graphs were made using Humann2's pathway abundance assignments derived originally from the Picrust analysis.

Qiime's group_significance.py was used to statistically compare OTU frequencies in sample groups using ANOVA. Qiime's group_significance.py was also used to compare functional predictions made by Picrust's categorize-by-function output. Qiime's group_significance.py was also used to compare pathway abundance predictions made by Humann2.

**Version of the software:**

Qiime: v1.9

FastQC: v0.11.5

Prinseq: v0.20.4

Krona: v2.7

Metaphlan2: v2017-03-03

Python3: v3.5.2

Matplotlib-venn 0.11.25

Graphlan: v0.9.7

R: v3.3.1

LEfSe: v2017

Picrust: v1.1.1

Humann2: v2

**Specific Programs Within a Software Package:**

gather.py -> Qiime: validate_mapping_file.py

fastqc.py -> FastQC:

trimPaired.py -> Prinseq:

fastqc.py -> FastQC:

JoinPairedEnd.py -> Qiime: join_paired_ends.py

SplitLibraries.py -> Qiime: split_libraries.py

Krona.py -> Krona:

ReadStats.py -> Prinseq:

ClosedRef.py -> Qiime: pick_closed_reference_otus.py

FilterOtus.py -> Qiime: filter_otus_from_otu_table.py

GroupSig.py -> Qiime: group_significance.py

Emperor.py -> Qiime: make_emperor.py

CompareCat.py -> Qiime: compare_categories.py

SummarizeTaxa.py -> Qiime: summarize_taxa_through_plots.py

MetaphlanHM.py -> Metaphlan2: metaphlan_hclust_heatmap.py

TaxVenn.py -> Python3:

graphlanQ.py -> Graphlan:

SampToTrt.py -> Python3:

sumtaxbox.py -> R:

LefseLDA.py -> LEfSe: format_input.py run_lefse.py plot_res.py plot_cladogram.py

picrust.py -> Picrust: normalize_by_copy_number.py predict_metagenomes.py categorize_by_function.py

SummarizeTaxa.py -> Qiime: summarize_taxa_through_plots.py

graphlanQPi.py -> Graphlan:

GroupSig.py -> Qiime: group_significance.py

preHsplit.py -> Humann2: humann2_split_table

graphlanQHu.py -> Graphlan:

GroupSig.py -> Qiime: group_significance.py

# Supplementary Figures

**Supplementary Figure 1.** Schematic illustration of the study design, showing order and duration of the rye kernel bread (RKB) or white wheat bread (WWB) consumption by participants (n=19) and total occasions for the stool collection (n=76) that also corresponds to the experimental day and blood collection at the research facility (n=76).

**Supplementary Figure 2.** Values of Shannon, Simpson indices and normal distribution plot of sequenced data **(A)**; microbiome alpha diversity (species richness) rarefactions for each group showed after as Chao1 estimation **(B)** and distance of observed species showed in unweighted unifraction PCoA plot **(C)**, where orange and green colors correspond to WWB 1d and 3d, respectively, while red and blue colors correspond to RKB 1d and 3d, respectively.

WWB, white wheat bread; RKB, rye kernel bread


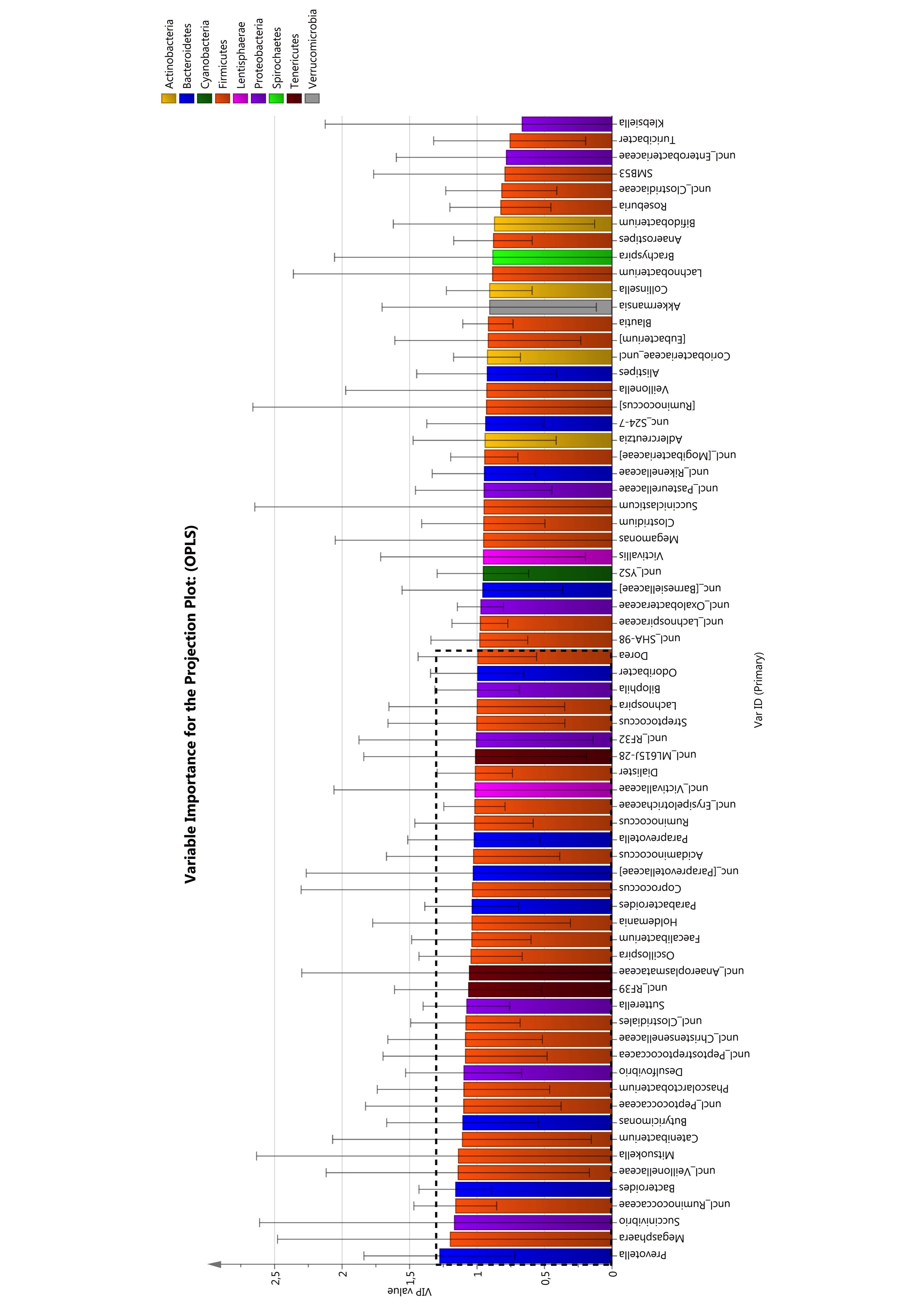
**Supplementary Figure 3.** The X variables (bacterial genera) of importance for the projection (VIP) are colored according to their taxonomic phylum and those variables that exceed VIP value >1 considered to be most significant in the prediction system (dashed rectangle).

**Supplementary Figure 4**. Permutation plots for the OPLS model showing R2 (green) and Q2 (blue) values

**References:**

Qiime: Schloss PD, Wescott SL, Ryabin T, Hall JR, Hartmann M, Hollister EB, Lesniewski RA, Oakley BB, Parks DH, Robinson CJ, Sahl JW, Stres B, Thallinger GG, Van Horn DJ, Weber CF. 2009. Introducing mothur: Open-source, platform-independent, community-supported software for describing and comparing microbial communities. Appl Environ Microbiol 75(23):7537-7541.

FastQC: <https://www.bioinformatics.babraham.ac.uk/projects/download.html#fastqc>

Prinseq: Schmieder R and Edwards R: Quality control and preprocessing of metagenomic datasets. Bioinformatics 2011, 27:863-864.

Krona: Ondov BD, Bergman NH, and Phillippy AM. Interactive metagenomic visualization in a Web browser. BMC Bioinformatics. 2011 Sep 30; 12(1):385.

Metaphlan2: Caporaso JG, Kuczynski J, Stombaugh J, Bittinger K, Bushman FD, Costello EK, Fierer N, Gonzalez Pena A, Goodrich JK, Gordon JI, Huttley GA, Kelley ST, Knights D, Koenig JE, Ley RE, Lozupone CA, McDonald D, Muegge BD, Pirrung M, Reeder J, Sevinsky JR, Turnbaugh PJ, Walters WA, Widmann J, Yatsunenko T, Zaneveld J, Knight R. 2010. QIIME allows analysis of high-throughput community sequencing data. Nature Methods 7(5): 335-336.

Python3: Vazquez-Baeza Y, Pirrung M, Gonzalez A, Knight R. 2013. Emperor: A tool for visualizing high-throughput microbial community data. Gigascience 2(1):16.

Graphlan: Caporaso JG, Kuczynski J, Stombaugh J, Bittinger K, Bushman FD, Costello EK, Fierer N, Gonzalez Pena A, Goodrich JK, Gordon JI, Huttley GA, Kelley ST, Knights D, Koenig JE, Ley RE, Lozupone CA, McDonald D, Muegge BD, Pirrung M, Reeder J, Sevinsky JR, Turnbaugh PJ, Walters WA, Widmann J, Yatsunenko T, Zaneveld J, Knight R. 2010. QIIME allows analysis of high-throughput community sequencing data. Nature Methods 7(5): 335-336.

R: Duy Tin Truong, Eric A Franzosa, Timothy L Tickle, Matthias Scholz, George Weingart, Edoardo Pasolli, Adrian Tett, Curtis Huttenhower & Nicola Segata. Nature Methods 12, 902-903 (2015)

LEfSe: <https://www.python.org/>

Picrust: <https://bitbucket.org/nsegata/graphlan>

Humann2: <https://github.com/picrust/picrust>
